# Profile and determinants of delayed care-seeking and diagnosis among patients with imported malaria: a retrospective study in China, 2014–2021

**DOI:** 10.1186/s40249-022-01050-3

**Published:** 2022-12-22

**Authors:** Tao Zhang, Duoquan Wang, Yingjun Qian, Wei Ruan, Ying Liu, Jing Xia, Hui Yan, Yuan Sui, Shenning Lu, Xian Xu, Jingjing Jiang, Xiaofeng Lyu, Shuqi Wang, Shizhu Li, Weidong Li

**Affiliations:** 1 Anhui Provincial Center for Disease Control and Prevention, Hefei, 230601 China; 2 National Institute of Parasitic Diseases, Chinese Center for Disease Control and Prevention (Chinese Center for Tropical Diseases Research), NHC Key Laboratory of Parasite and Vector Biology, WHO Collaborating Center for Tropical Diseases, National Center for International Research on Tropical Diseases, Shanghai, 200025 China; 3 Zhejiang Provincial Center for Disease Control and Prevention, Hangzhou, 310051 China; 4 Henan Provincial Center for Disease Control and Prevention, Zhengzhou, 450016 China; 5 Hubei Provincial Center for Disease Control and Prevention, Wuhan, 430079 China; 6 Guangxi Zhuang Autonomous Region Center for Disease Control and Prevention, Nanning, 530028 China; 7 Brown School, Washington University, St. Louis, MO USA; 8 School of Global Health, Chinese Center for Tropical Diseases Research, Shanghai Jiao Tong University School of Medicine, Shanghai, 200025 China

**Keywords:** Malaria, Delayed care-seeking, Delayed diagnosis, Imported malaria, Prevention, Re-establishment, China, *Plasmodium falciparum*

## Abstract

**Background:**

In areas where malaria has been eliminated, delayed care-seeking and diagnosis of imported malaria are constant threats. This study aimed to describe the profile and determinants of delayed care-seeking and diagnosis among patients with imported malaria in China.

**Methods:**

This retrospective study assessed surveillance data obtained from 2014 to 2021 in the Chinese provincial-level administrative divisions (PLADs) of Anhui, Henan, Hubei, and Zhejiang, and Guangxi. Epidemiological characteristics were analyzed using descriptive statistics. Furthermore, factors associated with delayed care-seeking and diagnosis among imported malaria cases were identified using multivariate logistic regression.

**Results:**

Overall, 11.81% and 30.08% of imported malaria cases had delays in seeking care and diagnosis, respectively. During the study period, there was a decreasing trend in the proportion of imported malaria cases with delayed care-seeking (*χ*^2^ = 36.099, *P *< 0.001) and diagnosis (*χ*^2^ = 11.395, *P =* 0.001). In multivariate analysis, independent risk factors associated with delayed care-seeking include PLADs (Guangxi as reference), consultations in high-level facilities for the first medical visit, infections with non-*Plasmodium falciparum* species, and older age. However, PLADs (Guangxi as reference), the purpose of traveling (labour as reference), and infections with non-*P. falciparum* species increased the risk of delayed diagnosis. Delayed care-seeking (adjusted odds ratio: 1.79, *P* = 0.001) and diagnosis (adjusted odds ratio: 1.62, *P* = 0.004) were risk factors for severe disease development.

**Conclusions:**

Based on this study’s findings, we strongly advocate for improved access to quality healthcare to reduce the rate of misdiagnosis at the first visit. Infections caused by non-*P. falciparum* species should be highlighted, and more sensitive and specific point-of-care detection methods for non-*P. falciparum* species should be developed and implemented. In addition, education programs should be enhanced to reach target populations at risk of malaria infection. All these factors may reduce delayed care-seeking and diagnosis of imported malaria.

**Graphical Abstract:**

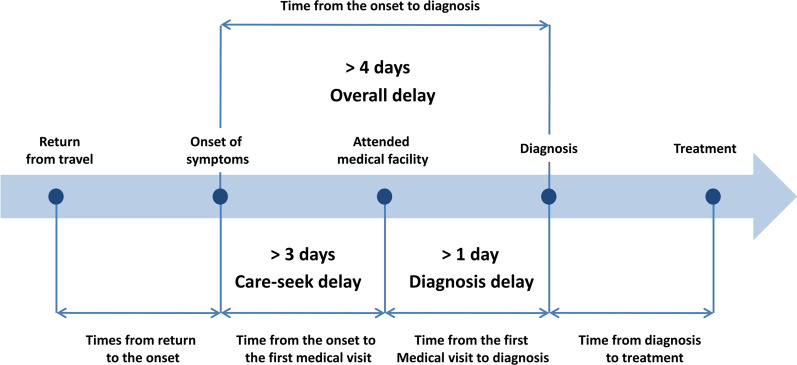

**Supplementary Information:**

The online version contains supplementary material available at 10.1186/s40249-022-01050-3.

## Background

Remarkable progress has been made in malaria elimination; however, malaria remains a serious public health problem worldwide, with populations in 85 endemic countries at risk [1]. The number of malaria cases increased from 227 million in 2019 to an estimated 241 million in 2020 due to disruptions in malaria control services during the coronavirus disease 2019 (COVID-19) pandemic. In addition, the estimated number of malaria deaths was 627,000, a 12% increase compared to that in 2019, with most of this increase occurring in countries in the World Health Organization (WHO) African Region [1]. Therefore, countries certified by the WHO as malaria-free—meaning that local transmission has been interrupted—such as China, should make concerted efforts to prevent the re-establishment of malaria transmission and guarantee global elimination thereof.

In China, the National Malaria Elimination Action Plan was launched in 2010 to eliminate malaria nationally by 2020 [2, 3]. Significant progress has been made under the guidance of the National Malaria Elimination Action Plan and the “1-3-7” work norms of surveillance and response [4], and the last indigenous cases were reported in Yunnan Province in 2016 [5]. The WHO declared China malaria-free on June 30, 2021, a milestone in global malaria elimination and Chinese public health [6]. However, due to imported malaria in China, maintaining malaria-free status is a constant challenge.

China has been under tremendous pressure from ‘imported’ malaria, that is, the infections acquired outside the country over the years [7]. Similar to other non-endemic countries, the pressure commonly starts with a delay in managing imported malaria cases, which is a leading cause of death and increases the risk of secondary local transmission [8–10]. According to a recent review by Bastaki et al., the delay in diagnosing imported malaria cases is primarily due to patients’ failure to seek medical care promptly and healthcare providers’ lack of diagnostic ability and awareness [11]. However, studies currently focusing on these two types of delays are still limited. Therefore, this study aimed to describe the profile and determinants of delayed care-seeking and diagnosis among patients with imported malaria in China.

This retrospective study used surveillance data from 2014 to 2021 in five Chinese provincial-level administrative divisions (PLADs) to describe profiles of delays and to identify predictors of delayed care-seeking (DC) and delayed diagnosis (DD) of imported malaria cases. These findings might contribute to developing innovative interventions for the prevention of re-establishment of malaria, which may improve early healthcare-seeking behavior, prompt diagnosis, and treatment of imported malaria cases.

## Methods

### Study setting

The provinces of Anhui, Henan, Hubei, and Zhejiang, and Guangxi Zhuang Autonomous Region, China, were selected for this study due to their burden of imported cases and risk of re-establishment (Additional file 1: Appendix S1). Historically, malaria was highly prevalent in the provinces of Anhui, Henan, and Hubei [12–14], which means high receptivity, namely, a relatively high risk of re-establishment of malaria. In Zhejiang, one of the most developed provinces in the country, the number of reported imported malaria cases has increased due to frequent international communication [15]. Guangxi is a border PLAD in southern China, where numerous migrant workers are exported to Africa. Therefore, the number of imported malaria cases in Guangxi is among the highest in China [16].

### Definition of imported malaria case, DC, and DD

Imported malaria case: malaria infection in a person whose presence of malaria parasites in the blood has been confirmed by a diagnostic test and the infection acquired outside the country (in this study, China) where it was diagnosed [17]. Severe malaria: defined according to the guidelines established by the National Health and Family Planning Commission of China [18]. It refers to confirmed cases with one or more of the following features: impaired consciousness or coma; severe anemia; renal impairment; pulmonary edema; acute respiratory distress syndrome; or other emergencies requiring intensive care unit admission. In this study, blood samples were collected before antimalarial treatment in imported malaria cases. The diagnosis was confirmed using microscopic examination and polymerase chain reaction in the provincial malaria diagnostic reference laboratory, according to the malaria diagnostic criteria in China [18]. The presence or absence of *Plasmodium* spp. and the species present were recorded after the examination.

### Delayed care-seeking and delayed diagnosis

**Fig. 1:**
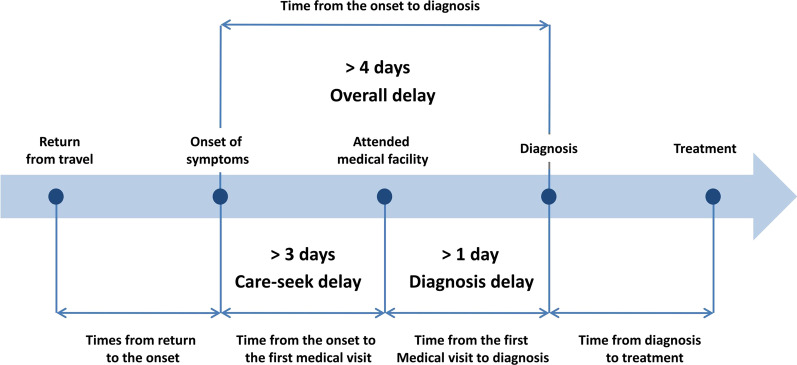
Definitions of delayed care-seeking and diagnosis in imported malaria

According to WHO guidelines, malaria should be promptly diagnosed and treated within 24 h of symptom onset to reduce the risk of severe disease and transmission [19]. In practice, it is difficult to achieve WHO’s recommendations in such a short period of time. In this study, a working definition reported by Bastaki et al. for overall delays (the interval between illness onset and diagnosis) greater than 4 d was employed [10]. Receiver operator characteristic (ROC) analysis was performed to test this working definition (Additional file 2: Appendix S2). The results showed an optimum cutoff value between 3.5 d and 4.5 d. Thus, the definition is appropriate. Furthermore, referring to previous research [20, 21], DC was defined as the interval between symptom onset and the first visit longer than 3 d, and DD was defined as the interval between the first consultation and malaria diagnosis longer than 1 d (Fig. 1). As antimalarial drugs are readily available in China, this study did not highlight treatment delays.

### Inclusion and exclusion criteria of the enrolled cases

This study included imported malaria cases reported in five PLADs from 2014 to 2021. Indigenous malaria cases were excluded. This study included 5765 imported malaria cases.

### Data source

This retrospective study evaluated surveillance data from January 2014 to December 2021 in five Chinese PLADs to identify the factors contributing to DC and DD. Malaria is a nationally notifiable disease in China, and each case must be reported to the Centers for Disease Control and Prevention through two reporting systems: the China Information System for Disease Control and Prevention (CISDCP) and the Information System for Parasitic Disease Control and Prevention (ISPDCP), a subsystem of the CISDCP. Moreover, staff from county-level Centers for Disease Control and Prevention must complete an epidemiological report to identify the origin of the infection and record other valuable information. Demographic, epidemiological, and clinical data of patients with malaria, including age, sex, occupation, travel history, date of symptom onset, diagnosis, treatment, symptoms, complications, and prognosis, were extracted from the CISDCP, ISPDCP, and epidemiological survey reports.

### Statistical analysis

Normally distributed quantitative variables were presented as means and standard deviations, whereas non-normally distributed variables were reported as medians and interquartile ranges (IQR). Categorical variables were expressed as percentages. Differences in proportions were assessed using Pearson’s chi-squared *χ*^2^ or Fisher’s exact test, as appropriate. The Bonferroni-corrected post-hoc test was conducted to adjust the observed significant level for multiple comparisons. The chi-square test for trend was used to describe the statistical significance of the differences between years. Factors associated with DC and DD were determined using hierarchical logistic regression analysis. The association between delays and exposure variables was estimated using odds ratios (*OR*s) and 95% confidence intervals (*CI*s). In the first layer, only one variable (PLAD) was included. In the second layer, variables considered significant (*P* < 0.1) in the univariate analysis were included in the multivariable model, and non-significant variables were removed using a forward stepwise approach. Only significant variables (*P* < 0.05) were included in the final model. Forest plots were used to display the results of multivariate logistic regression. Imputation for missing data was addressed using the dummy variable method. Statistical significance was set at *P* < 0.05. ROC analysis (with severe malaria as an outcome variable and time between symptom onset and diagnosis as a predictor) was used to determine the optimal cut-off value for this study. All statistical tests were performed using SPSS version 26.0 (SPSS Inc., Chicago, IL, USA). ROC curve analysis was performed using MedCalc statistical software version 20.115 (MedCalc Software Ltd, Ostend, Belgium).

## Results

### Epidemiological profile

This study included 5765 imported malaria cases (743 in Anhui, 1284 in Zhejiang, 832 in Hubei, 1643 in Guangxi, and 1263 in Henan), and the annual number of cases from 2014 to 2021 ranged from 139 to 1035. The number of infections peaked in 2016 at 1035 (17.95%). Only 139 cases were reported in 2021 because of travel restrictions due to the COVID-19 pandemic. The mean age of all patients was 41.11 ± 9.92 years, and 5532 (95.96%) patients were males. Migrant laborers comprised 81.53% (4700/5765) of the patients. Most infections (5536, 96.03%) originated in Africa, especially Ghana (854, 14.81%), Nigeria (699, 12.12%), Cameroon (565, 9.80%), Angola (553, 9.59%), and the Democratic Republic of the Congo (446, 7.74%). The predominant species was *Plasmodium falciparum* (3996 cases, 69.31%), followed by *P. ovale* (1067, 18.51%), *P. vivax* (416, 7.22%), *P. malariae* (203, 3.52%), and *P. knowlesi* (1, 0.02%). There were 82 mixed-species infections (1.42%). During the study period, 297 patients (5.15%) developed severe malaria, leading to 20 deaths (four in Anhui, eight in Henan, three in Hubei, three in Guangxi, and two in Zhejiang), with a fatality rate of 0.35%. Table 1 summarizes the epidemiological characteristics of imported malaria cases.

**Table 1:** Epidemiological characteristics of imported malaria cases in China from 2014 to 2021

Characteristics (*n* = 5765)	Number (%)
Sex	
Male	5532 (95.96)
Female	233 (4.04)
Age (years), mean ± SD	41.11 ± 9.92
Purpose of travel	
Labor	4700 (81.53)
Official duties	248 (4.30)
Business	669 (11.60)
Others	148 (2.57)
Origin of infection	
Africa	5536 (96.03)
Asia	207 (3.59)
Oceania	14 (0.24)
South America	8 (0.14)
Malaria species	
* Plasmodium falciparum*	3996 (69.31)
* P. ovale*	1067 (18.51)
* P. vivax*	416 (7.22)
* P. malariae*	203 (3.52)
* P. knowlesi*	1 (0.02)
Mixed infections	82 (1.42)

*SD* standard deviation

### Healthcare seeking behavior

At the first consultation, the number of patients who received care in PLAD, city, county, township, and private/village health centers was 627 (10.88%), 1314 (22.79%), 2724 (47.25%), 278 (4.82%), and 697 (12.09%), respectively. One hundred and twenty-five patients (2.17%) sought other health services (overseas hospitals, inspection, and quarantine institutions). In addition, 1478 patients (25.64%) were misdiagnosed during their first visit. Misdiagnosis rates in private/village and township health centers were 91.68% (639/697) and 49.64% (138/278), respectively (Additional file 3: Appendix S3). Additional file 4: Appendix S4 presents the detailed results of the first healthcare seeking among patients with imported malaria in the five PLADs.

### Delayed care-seeking, delayed diagnosis, and overall delay

The median number of days from the date of arrival in China to symptom onset was 9 d (IQR: 3–29 d). Among 4795 cases with information on the date of arrival in China, 3648 (76.08%) experienced symptoms within 30 d. The median interval between symptom onset and the first visit was 1 d (IQR: 0–3 d), and 5084 (88.19%) patients sought medical care within 3 d. The median time between the first visit and diagnosis was 1 d (IQR: 0–2 d), and 4031 (69.92%) cases were correctly diagnosed within 1 d of the first visit. The median time between symptom onset and diagnosis was 2 d (IQR: 1–5 d), and 4228 cases were diagnosed within 4 d after symptom onset. During the study period, the proportions of DC (*χ*^2^ = 36.099, *P* < 0.001) and DD (*χ*^2^ = 11.395, *P =* 0.001) decreased (Additional file 5: Appendix S5).

The proportion of DC among imported patients in Anhui, Guangxi, Zhejiang, Hubei, and Henan was 13.06%, 10.59%, 12.46%, 13.10%, and 12.19%, respectively. For DD, the proportions were 37.28%, 13.02%, 27.08%, 39.42%, and 35.47%, respectively. For the overall delay, the proportions were 34.05%, 15.52%, 24.38%, 35.82%, and 33.10%, respectively. Post-hoc multiple comparison tests were performed to confirm the differences between PLADs. The results revealed that Guangxi had a lower proportion of DC than Zhejiang, Hubei, Anhui, and Henan. However, Guangxi had a lower proportion of DD than Anhui, Zhejiang, Hubei, and Henan, which is consistent with the overall delay situation (see Table 2).

**Table 2:** Time between symptom onset and diagnosis of imported malaria cases in China, 2014–2021

PLAD	Days from onset to first medical visit		Days from first medical visit to diagnosis		Days from onset to diagnosis	
	≤ 3 d	> 3 d	≤ 1 d	> 1 d	≤ 4 d	> 4 d
Guangxi	1503 (91.48)	140 (8.52)^a^	1429 (86.98)	214 (13.02)^a^	1388 (84.48)	255 (15.52)^a^
Zhejiang	1125 (87.62)	159 (12.38)^b^	927 (72.20)	357 (27.80)^b^	971 (75.62)	313 (24.38)^b^
Hubei	713 (85.70)	119 (14.30)^b^	447 (53.73)	385 (46.27)^c^	534 (64.18)	298 (35.82)^c^
Anhui	653 (87.89)	90 (12.11)^b^	413 (55.59)	330 (44.41)^c^	490 (65.95)	253 (34.05)^c^
Henan	1090 (86.30)	173 (13.70)^b^	815 (64.53)	448 (35.47)^d^	845 (66.90)	418 (33.10)^c^
*χ*^2^	26.815		424.193		190.89	
*P*-value	< 0.001		< 0.001		< 0.001	

*PLAD* provincial-level administrative division

^a, b, c, d^Different letter subscripts indicate statistical significance between subsets at a level of 0.05. *P*-values were adjusted for multiple comparisons using the Bonferroni correction

DC and DD were experienced by 11.81% (681 cases) and 30.08% (1734 cases) of the patients, respectively. Univariate analysis was performed to explore the potential contributors to these delays. The factors associated with DC included age, travel region, malaria species, level of healthcare facilities for the initial medical visit, and time between arrival in China and symptom onset. The purpose of travel, travel region, malaria species, level of healthcare facilities for the initial medical visit, diagnostic result in the first visit, and history of malaria infection were statistically associated with DD (Table 3).

**Table 3:** Characteristics of patients with imported malaria in China from 2014 to 2021

Characteristics	Delayed care-seeking				Delayed diagnosis			
	Yes, *n* (%)	No, *n* (%)	*χ*^2^	*P*-value	Yes, *n* (%)	No, *n* (%)	*χ*^2^	*P*-value
Sex			0.094	0.760			0.078	0.780
Male	652 (11.79)	4880 (88.21)			1662 (30.04)	3870 (69.96)		
Female	29 (12.45)	204 (87.55)			72 (30.90)	161 (69.10)		
Age (years)			10.892	0.012			7.016	0.071
< 30	99 (9.74)	917 (90.26)			335 (32.97)	681 (67.03)		
30–39	178 (10.74)	1480 (89.27)			467 (28.17)	1191 (71.83)		
40–49	267 (13.25)	1748 (86.75)			611 (30.32)	1404 (69.68)		
≥ 50	137 (12.73)	939 (87.27)			321 (29.83)	755 (70.17)		
Purpose of travel			1.938	0.585			33.377	< 0.001
Labor	562 (11.96)	4138 (88.04)			1363 (29.00)	3337 (71.00)		
Official duties	33 (13.31)	215 (86.69)			110 (44.35)	138 (55.65)		
Business	70 (10.46)	599 (89.54)			202 (30.19)	467 (69.81)		
Others	16 (10.81)	132 (89.19)			59 (39.86)	89 (60.14)		
Region of travel			14.064	< 0.001			17.099	< 0.001
African countries	636 (11.49)	4900 (88.51)			1637 (29.57)	3899 (70.43)		
Non-African countries	45 (19.65)	184 (80.35)			97 (42.36)	132 (57.64)		
Malaria species			40.623	< 0.001			7.828	0.020
*Plasmodium falciparum*	401 (10.04)	3595 (89.96)			1157 (28.95)	2839 (71.05)		
Non-*P. falciparum*	264 (15.65)	1423 (84.35)			550 (32.60)	1137 (67.40)		
Mixed-infections	16 (19.51)	66 (80.49)			27 (32.93)	55 (67.07)		
The level of health facilities for initial medical visit			47.552	< 0.001			1195.312	< 0.001
Private/village	44 (6.31)	654 (93.69)			565 (81.06)	132 (18.94)		
Township-level	30 (10.79)	248 (89.21)			140 (50.36)	138 (49.64)		
County-level	300 (11.01)	2424 (88.99)			434 (15.93)	2290 (84.07)		
City-level	212 (16.13)	1102 (83.87)			339 (25.80)	975 (74.20)		
Province-level	83 (13.24)	544 (86.76)			205 (32.70)	422 (67.30)		
Others	12 (9.60)	113 (90.40)			51 (40.80)	74 (59.20)		
Diagnosis at the first visit	NA		NA				2365.492	< 0.001
Other diseases					1184 (80.11)	294 (19.89)		
Malaria					550 (12.83)	3737 (87.17)		
Time from arrival in China to symptom onset (days)^a^			10.476	0.005			1.778	0.411
≤ 30 days	393 (10.77)	3255 (89.23)			1118 (30.65)	2530 (69.35)		
> 30 days	153 (13.34)	994 (86.66)			339 (29.56)	808 (70.44)		
Missing	135 (13.92)	835 (86.08)			277 (28.56)	693 (71.44)		
History of malaria infection			0.611	0.435			103.039	< 0.001
No	205 (12.33)	1457 (87.67)			660 (39.71)	1002 (60.29)		
Yes	476 (11.60)	3267 (88.40)			1074 (26.18)	3029 (73.82)		

*NA* not applicable

^a^4795 patients (83.17%) with available information.

**Fig. 2:**
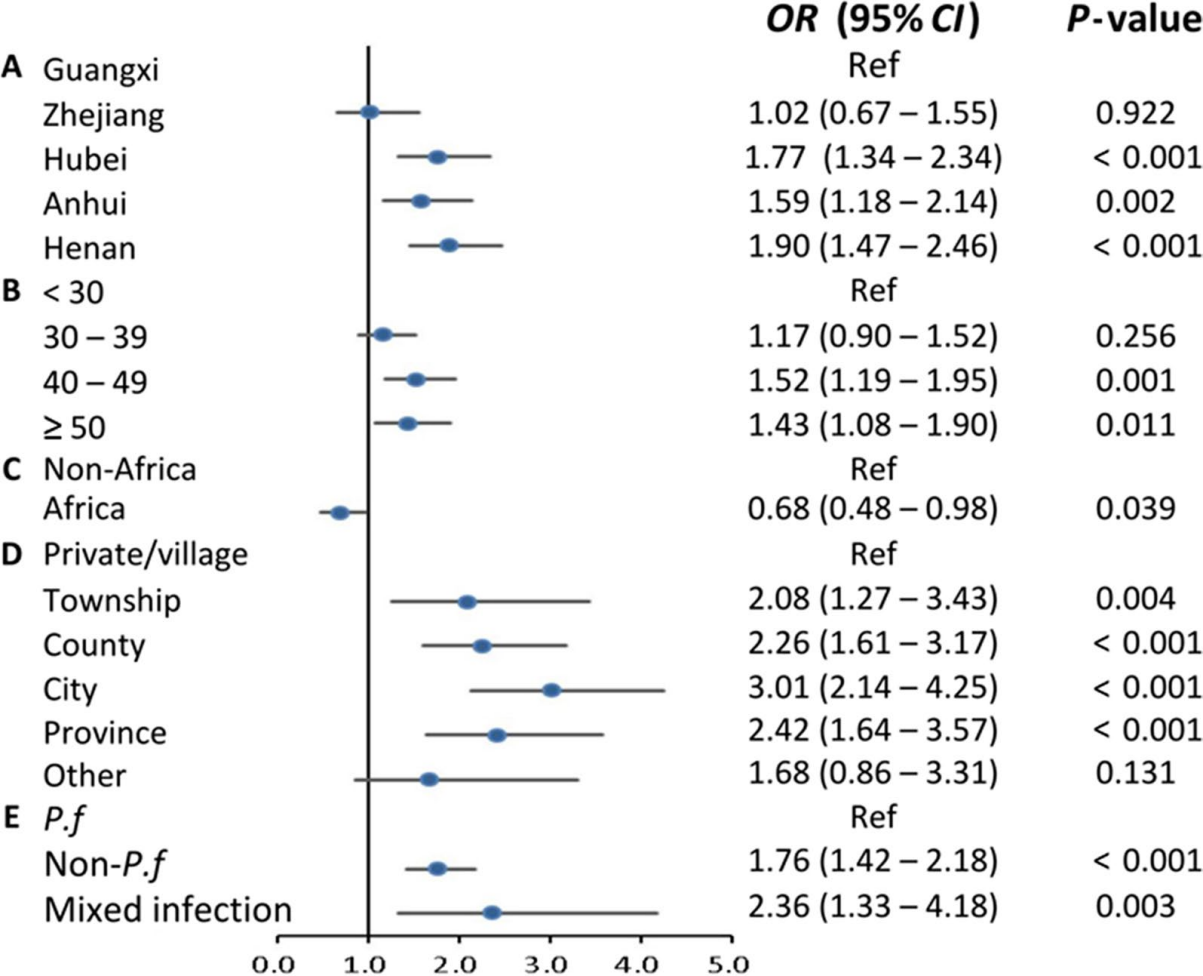
Factors associated with delayed care-seeking among imported malaria patients in China, 2014–2021. **A** PLADs; **B** age (years); **C** regions; **D** the level of health facilities for initial medical visit; **E** malaria species. *CIs* confidence intervals, *ORs* odds ratios, *PLADs* provincial-level administrative divisions, *P.f* *Plasmodium falciparum*

**Fig. 3:**
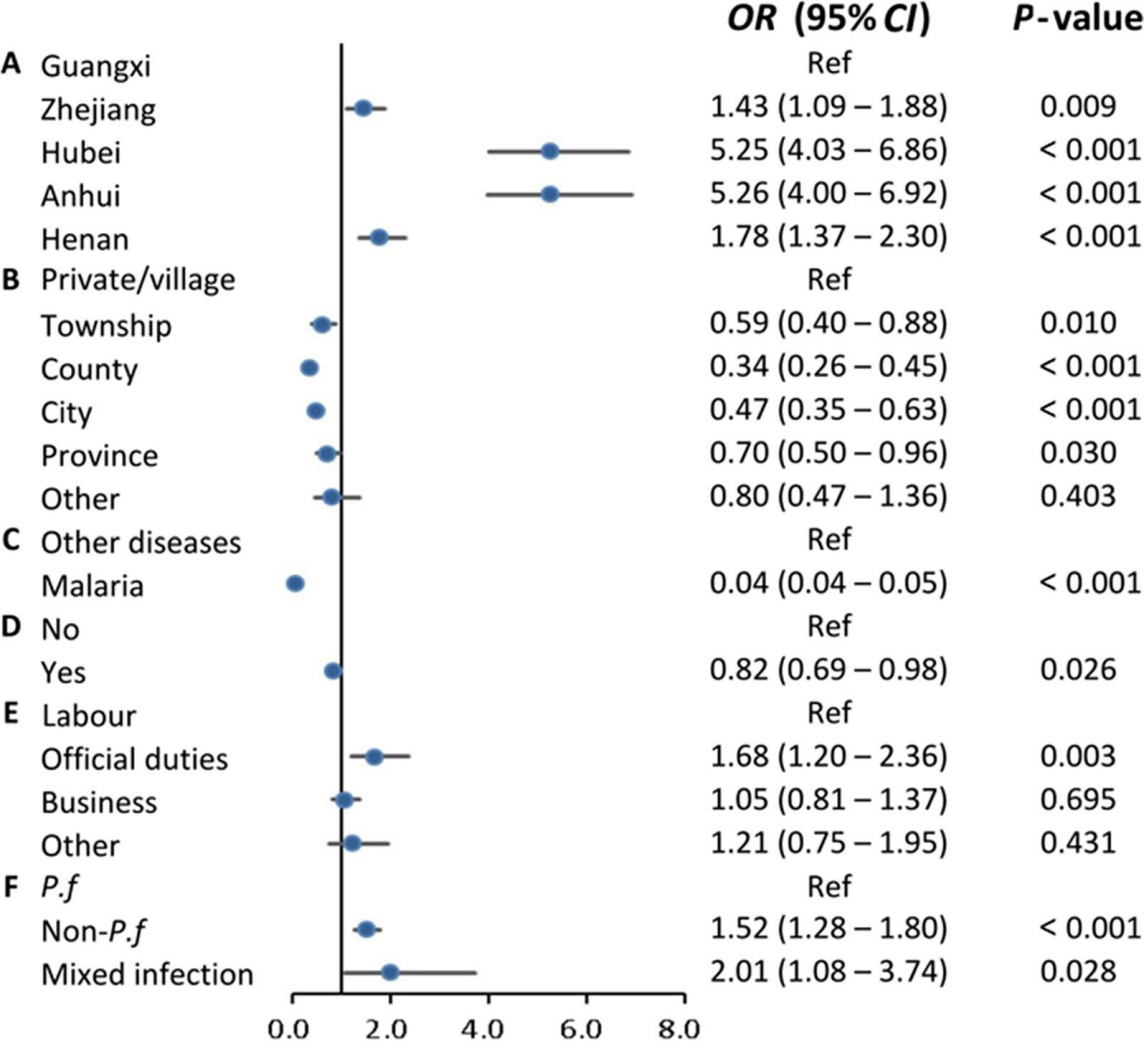
Factors associated with delayed diagnosis among imported malaria patients in China, 2014–2021. **A** PLADs; **B** the level of health facilities for initial medical visit; **C** the diagnosis result of first medical visit; **D** history of infection; **E** purpose of travel; **F** malaria species. *CIs* confidence intervals, *ORs* odds ratios, *PLADs* provincial-level administrative divisions, *P.f* *Plasmodium falciparum*

Considering the differences between PLADs, multivariate hierarchical logistic regression was used to identify the factors influencing DC and DD. The results indicated that the independent risk factors associated with DC were PLADs (Zhejiang, Hubei, Anhui, and Henan vs Guangxi), older age, infections with non-*P. falciparum* species, and consultations in high-level facilities for the first medical visit. Conversely, compared to other regions, patients returning from Africa seek medical care more promptly. PLADs (Zhejiang, Hubei, Anhui, and Henan vs Guangxi), the purpose of travel (official duties and business vs labor), and infections with non-*P. falciparum* species increased the risk of DD. In contrast, consultations in high-level health facilities for the initial medical visit, correct diagnosis at the first visit, and history of infection were conducive to timely diagnosis. Figures 2 and 3 depict the results of the multivariate regression analysis.

### Association between delays and severe malaria

The association between delays and severe malaria were also assessed. The crude *OR*s for the DC, DD, and overall delay associated with severe malaria were 1.59, 3.44, and 2.68, respectively. After adjusting for covariates, DC (adjusted *OR* = 1.79, 95% *CI* 1.29–2.50), DD (adjusted *OR* = 1.62, 95% *CI* 1.70–2.26), and overall delay (adjusted *OR* = 1.89, 95% *CI* 1.45–2.45) increased the risk of severe malaria (Table 4).

**Table 4:** Crude and adjusted odds ratios for the association of care-seeking and diagnosis delays with severe malaria

Factor	Rate of severe illness, *n*/*N* (%)	Crude odds ratio (95% *CI*)	*P*-value	Adjusted odds ratio (95% *CI*)	*P*-value
Delayed care-seeking					
No	246/5084 (4.84)	1.59 (1.16–2.17)	0.004	1.79 (1.29–2.50)^a^	0.001
Yes	51/681 (7.48)				
Delayed diagnosis					
No	125/4031 (3.10)	3.44 (2.72–4.37)	< 0.001	1.62 (1.70–2.26)^b^	0.004
Yes	172/1734 (9.92)				
Overall delay					
No	155/4228 (3.67)	2.68 (2.12–3.39)	< 0.001	1.89 (1.45–2.45)^b^	< 0.001
Yes	142/1537 (9.24)				

*n*: the number of severe cases with delayed care-seeking, delayed diagnosis, and overall delay, respectively. *N*: the number of imported cases with delayed care-seeking, delayed diagnosis, and overall delay, respectively

*CI* confidence interva, *PLAD* provincial-level administrative division

^a^Adjusted for age, sex, region of infection acquisition, level of health facilities for initial medical visit, history of malaria infection, time from return to China to onset of illness, purpose of travel, PLAD, and species

^b^Adjusted for age, sex, region of infection acquisition, level of health facilities for initial medical visit, diagnosis result of first medical visit, history of malaria infection, time from return to China to onset of illness, purpose of travel, PLAD, and species

## Discussion

In China, local malaria transmission was interrupted since 2017 [5], and prevention of re-establishment of malaria became a high priority. Therefore, imported malaria cases should be closely monitored. However, delays in managing imported malaria caused by patient and medical factors [10] are common in non-endemic areas, potentially increasing the risk of severe malaria and local transmission. In our study, 11.81% and 30.08% of the patients experienced DC and DD, respectively. Nevertheless, DC and DD rates decreased over the study period, suggesting improved early healthcare-seeking behavior and prompt diagnosis. Further analysis indicated that DC (adjusted *OR* = 1.79, 95% *CI* 1.29–2.50) and DD (adjusted *OR* = 1.62, 95% *CI* 1.70–2.26) significantly contributed to severe disease development. This is consistent with previous findings that a 4–12 d delay in diagnosis increases the risk of developing severe malaria [22]. Therefore, identifying and addressing the factors associated with DC and DD could improve the management of imported malaria cases.

Early healthcare-seeking behavior, prompt diagnosis, and treatment were consistent pursuits; however, there were differences in managing imported malaria across the PLADs studied. Guangxi had the best performance in terms of care seeking and diagnosis among imported malaria cases, followed by Zhejiang. Subsequently, Anhui, Hubei, and Henan came close in performance. One possible explanation is the epidemiological profile differences between the PLADs. For example, 85.43% of malaria cases were reported in Nanning City (the capital of the Guangxi Zhuang Autonomous Region), with high spatial aggregation [23]. In 2013, a large number of gold miners returned from Ghana, resulting in an outbreak of imported malaria (> 1000 cases) in Shanglin County, Nanning City [24]. All these factors promoted the awareness and diagnostic ability of medical institutions in Guangxi, especially at the epicenter of Nanning City. Zhejiang has a different story; this province is one of the most developed in the country with the best medical facilities. In contrast, the provinces of Anhui, Hubei, and Henan do not have abundant medical resources. Similarly, imported malaria cases were widely distributed across these three provinces [25–27]. Therefore, improving prompt diagnosis and treatment of imported malaria cases in the provinces of Anhui, Hubei, and Henan is a significant challenge.

In this study, multivariate logistic regression models were used to identify factors influencing DC and DD to guide prevention of re-establishment of malaria practice better. Obtaining care in higher-level health facilities on the first medical visit and non-*P. falciparum* species were risk factors for DC and DD. During the research, 80.92% (4665/5765) of patients sought care in county health facilities or higher at the first medical visit, in line with previous studies [21, 28]. These preferences reflected the common perception that higher-level health facilities could provide high-quality medical services while primary healthcare institutions were inadequate, which is accurate, at least for malaria diagnosis. Our data showed that the misdiagnosis rate at the first visit was 91.68% in private/village clinics, 49.64% in primary hospitals, and 14.23% in county and other higher-level centers. This implies that patients receiving care in primary health facilities were commonly misdiagnosed, leading to DD. In contrast, according to our findings, primary health facilities can improve access to health care, decreasing the risk of DC. This is because most malaria cases occur in Chinese migrant workers living in remote rural areas [29, 30], where access to quality healthcare is limited. Thus, primary healthcare institutions, including village and private clinics and township hospitals, are more accessible but have limited medical resources [25, 31]. This seems contradictory, and a proper balance must be achieved. Therefore, we strongly recommend that primary health facilities maintain vigilance and refer suspected patients to high-level hospitals for diagnosis and treatment.

In contrast, infections with non-*P. falciparum* species contributed to DC and DD. The following are possible explanations. First, these infections are more benign than *P. falciparum* infections, which can progress rapidly to severe forms of the disease [32]. Second, traditional methods for diagnosing malaria, such as light microscopy and malaria rapid diagnostic tests, have low specificity or sensitivity to non-*P. falciparum* spp. [33]. Third, malaria symptoms, especially for non-*P. falciparum* species, can develop 1 to 2 years after infection [34–36]. All these factors make the detection of non-*P. falciparum* infections difficult. Non-*P. falciparum* infections can cause severe malaria and, occasionally, even death [37, 38]. Another concern is the risk of malaria caused by *P. vivax*, whose vector is widespread in China [39]. The DD of imported *P. vivax* cases increases the risk of local transmission [36]. This is because gametocytes of *P. vivax* emerge at an early stage of infection, before onset of illness [37]. Additionally, during the COVID-19 pandemic, a higher proportion of non-*P. falciparum* infections were reported [40]. The above evidence underscores the need to focus on infections caused by non-*P. falciparum* species; therefore, more sensitive and specific point-of-care detection methods for non-*P. falciparum* species must be developed and implemented.

Prompt diagnosis and treatment of malaria are fundamental to adequate disease management. This study revealed that a correct diagnosis at the first visit was beneficial for timely diagnosis. This underlines the importance of the first diagnosis responsibility, requiring an improvement in clinicians’ awareness of malaria epidemiology, diagnosis, and treatment. A history of infection was another protective factor against DD. This was because patients previously infected with malaria were more likely to report a travel history, possibly due to an increased awareness of symptoms. In contrast, patients without a previous infection tend to self-treat their symptoms [41]. Interestingly, patients traveling for other purposes were at a higher risk of DD than migrant workers were. This finding is similar to another finding that patients returning from Africa seek medical care more promptly. These findings have important implications for extensive educational programs aimed at malaria-prone populations. Moreover, effective cooperation between disease control, customs, labor, and business departments is necessary to increase malaria infection awareness among at-risk populations and timely screening of suspected cases.

The retrospective nature of this study may have led to recall bias. In addition, healthcare-seeking behavior among patients and malaria prevention and control programs may have affected measures during the COVID-19 pandemic. Finally, the practice of prevention of re-establishment of malaria in border regions is more complex. The findings of this study may not be directly applicable to these regions.

## Conclusions

Based on this study’s findings, we strongly recommend improving access to quality healthcare to reduce misdiagnosis rate during the first visit. Infections caused by non-*P. falciparum* species should be highlighted, and more sensitive and specific point-of-care detection methods for non-*P. falciparum* species must be developed and implemented. In addition, educational programs should be enhanced to reach target populations at risk of malaria infection. All these factors may improve DC and DD among imported malaria cases.

## Supplementary Information

**Additional file 1: Appendix S1.** Location of the study area in China.

**Additional file 2: Appendix S2.** The ROC analysis between severe malaria and the time between symptom onset and diagnosis.

**Additional file 3: Appendix S3.** Diagnostic results of imported malaria patients at their first medical visits by healthcare facility levels.

**Additional file 4: Appendix S4.** Diagnostic accuracy at the first medical visit among imported malaria cases in China from 2014 to 2021.

**Additional file 5: Appendix S5.** Delayed care-seeking and diagnosis among imported malaria in China, 2014–2021.

## Data Availability

The dataset analyzed during the current study are available from the corresponding authors on reasonable request.

## Abbreviations

*CI*s: Confidence intervals
CISDCP: China Information System for Disease Control and Prevention
COVID-19: Coronavirus disease 2019
DC: Delayed care-seeking
DD: Delayed diagnosis
ISPDCP: Information System for Parasitic Disease Control and Prevention
*OR*s: Odds ratios
PLADs: Provincial-level administrative divisions
WHO: World Health Organization
