# Supplementary material for: Profile and determinants of delayed care-seeking and diagnosis among patients with imported malaria: a retrospective study in China, 2014–2021
Source: Infect Dis Poverty. 2022 Dec 22;11:125. doi: 10.1186/s40249-022-01050-3 (PMC9773583; doi:10.1186/s40249-022-01050-3)
Supplement: Supplementary file 1 — Additional file 1: Appendix S1. Location of the study area in China. [file 40249_2022_1050_MOESM1_ESM.docx]

**
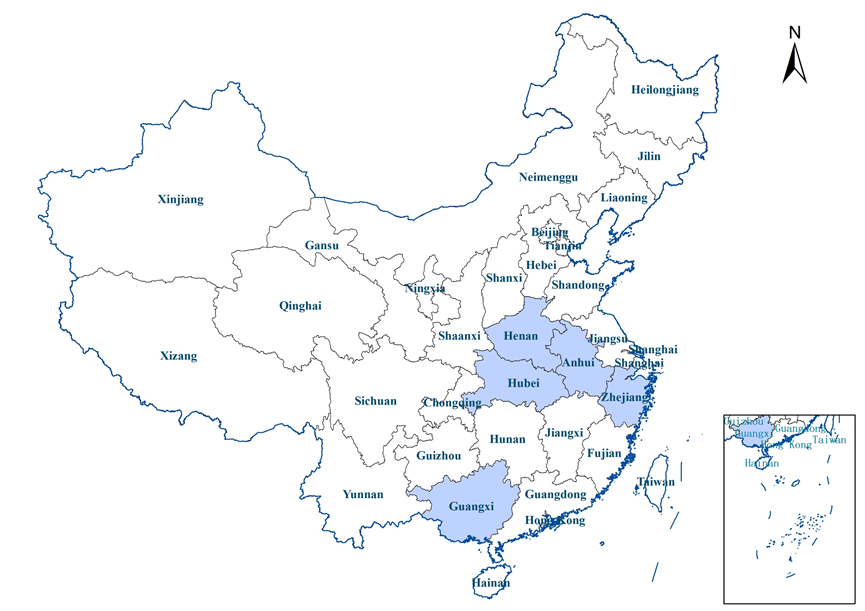
**

**Location of the study area in China.**

The thematic map of geographic distribution was compiled using MapInfo 15.0 (Pitney Bowes Inc., Troy, NY, USA).
