# Supplementary figures and images for: Profile and determinants of delayed care-seeking and diagnosis among patients with imported malaria: a retrospective study in China, 2014–2021
Source: Infect Dis Poverty. 2022 Dec 22;11:125. doi: 10.1186/s40249-022-01050-3 (PMC9773583; doi:10.1186/s40249-022-01050-3)

**
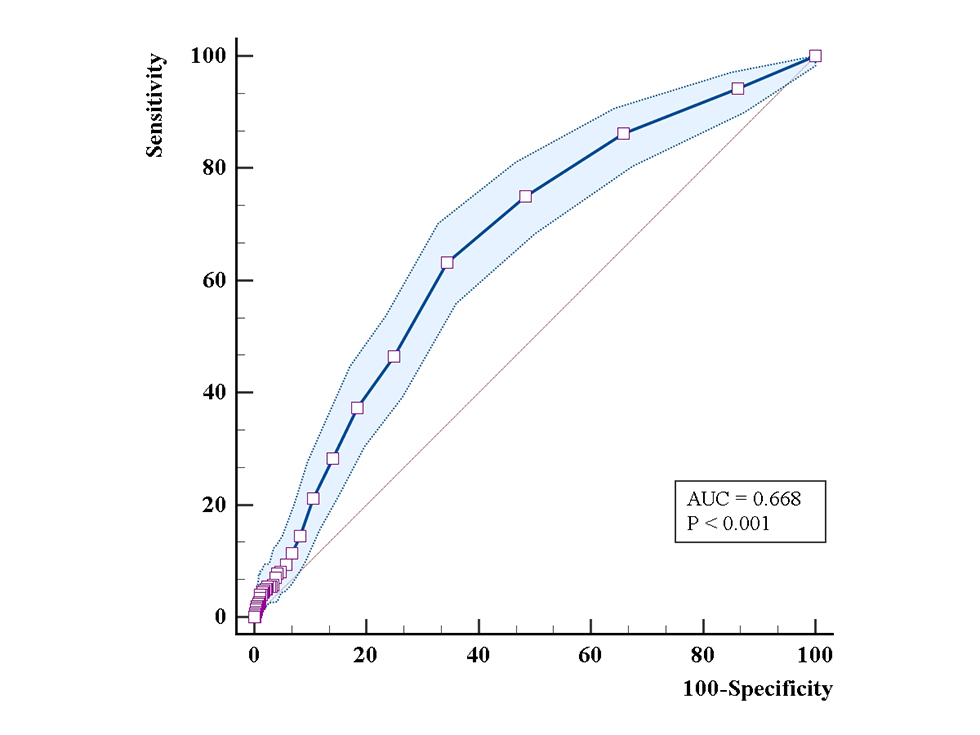
**

Supplement: Supplementary file 2 — Additional file 2: Appendix S2. The ROC analysis between severe malaria and the time between symptom onset and diagnosis. [file 40249_2022_1050_MOESM2_ESM.docx]

**
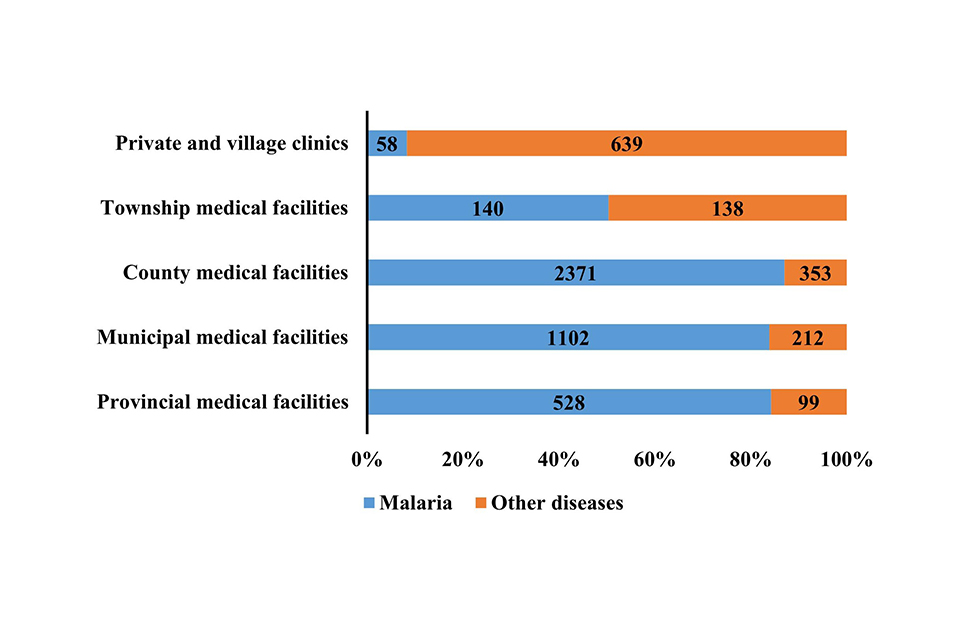
**

Supplement: Supplementary file 3 — Additional file 3: Appendix S3. Diagnostic results of imported malaria patients at their first medical visits by healthcare facility levels. [file 40249_2022_1050_MOESM3_ESM.docx]

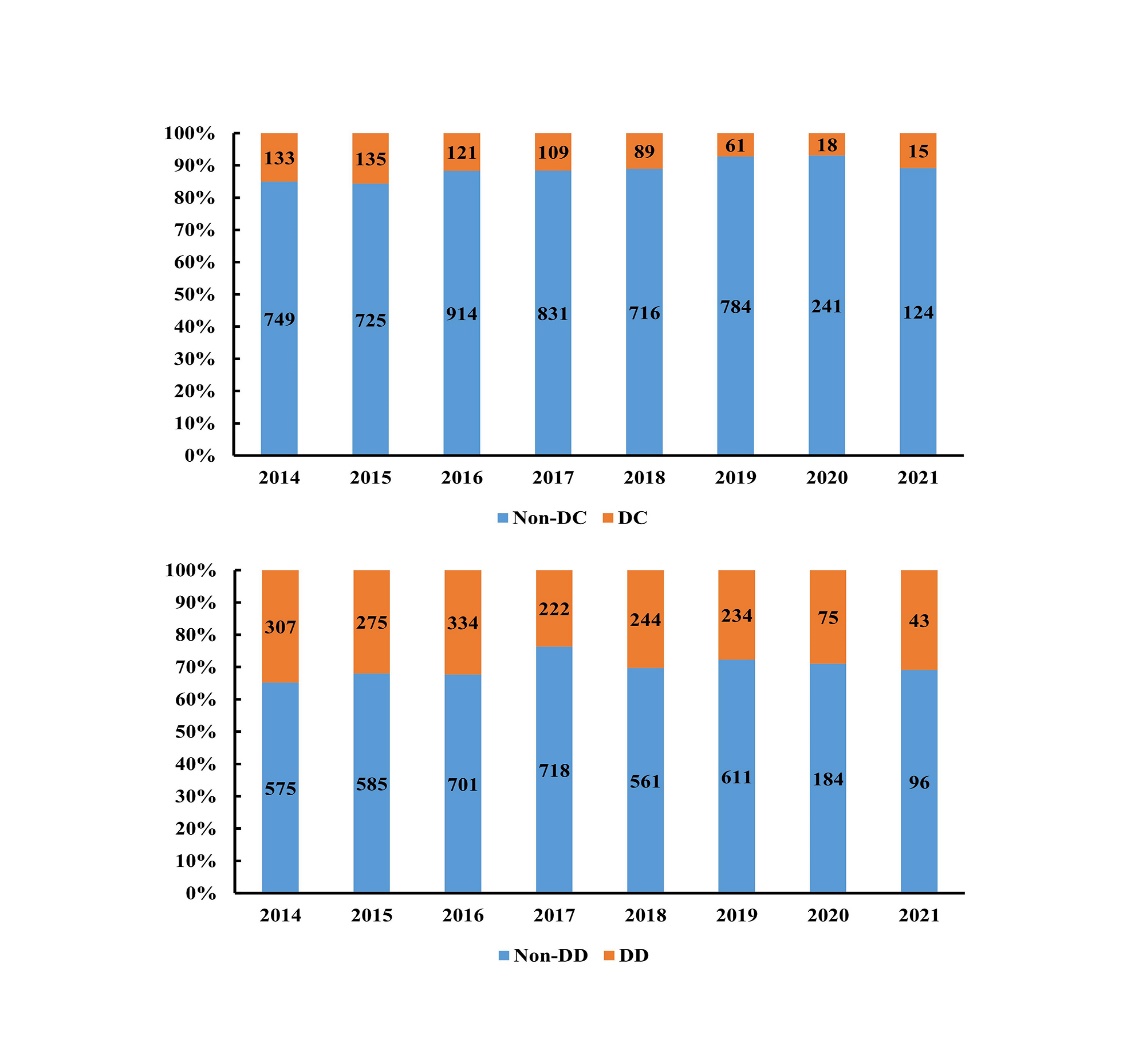

Supplement: Supplementary file 5 — Additional file 5: Appendix S5. Delayed care-seeking and diagnosis among imported malaria in China, 2014–2021. [file 40249_2022_1050_MOESM5_ESM.docx]
