# Supplementary material for: Profile and determinants of delayed care-seeking and diagnosis among patients with imported malaria: a retrospective study in China, 2014–2021
Source: Infect Dis Poverty. 2022 Dec 22;11:125. doi: 10.1186/s40249-022-01050-3 (PMC9773583; doi:10.1186/s40249-022-01050-3)
Supplement: Supplementary file 4 — Additional file 4: Appendix S4. Diagnostic accuracy at the first medical visit among imported malaria cases in China from 2014 to 2021. [file 40249_2022_1050_MOESM4_ESM.docx]

**Appendix S4 Diagnostic accuracy at the first medical visit among imported malaria cases in China from 2014 to 2021.**

| **Provinces** | **Level of health facilities for initial medical visit** | **Diagnosis result of ﬁrst medical visit** | | **Diagnostic accuracy (%)** |
| --- | --- | --- | --- | --- |
|  |  | **Malaria (*n*)** | **Other diseases (*n*)** |  |
| Guangxi | Private and village clinics | 8 | 63 | 11.27 |
|  | Township-level | 68 | 39 | 63.55 |
|  | County-level | 1202 | 50 | 96.01 |
|  | City-level | 126 | 23 | 84.56 |
|  | Province-level | 23 | 22 | 51.11 |
| Zhejiang | Private and village clinics | 21 | 170 | 10.99 |
|  | County-level | 487 | 140 | 77.67 |
|  | City-level | 248 | 53 | 82.39 |
|  | Province -level | 113 | 13 | 89.68 |
| Hubei | Private and village clinics | 9 | 68 | 11.69 |
|  | Township-level | 33 | 45 | 42.31 |
|  | County-level | 210 | 57 | 78.65 |
|  | City-level | 240 | 50 | 82.76 |
|  | Province-level | 82 | 28 | 74.55 |
| Anhui | Private and village clinics | 9 | 88 | 9.28 |
|  | Township-level | 32 | 25 | 56.14 |
|  | County-level | 187 | 29 | 86.57 |
|  | City-level | 158 | 26 | 85.87 |
|  | Province-level | 139 | 16 | 89.68 |
| Henan | Private and village clinics | 11 | 250 | 4.21 |
|  | Township-level | 7 | 29 | 19.44 |
|  | County-level | 285 | 77 | 78.73 |
|  | City-level | 330 | 60 | 84.62 |
|  | Province-level | 171 | 20 | 89.53 |
